# Nutrition education to type 1 diabetes patients: few changes over the time

**DOI:** 10.3389/fcdhc.2023.1243237

**Published:** 2023-08-29

**Authors:** Aurélien Clerc

**Affiliations:** ^1^ Nutrition Unit, University Training Hospital, Fribourg, Switzerland; ^2^ Department of Nutrition and Dietetics, School of Health Sciences, University of Applied Sciences and Arts Western Switzerland (HES-SO), Geneva, Switzerland

**Keywords:** type 1 diabetes, diabetes nutrition, diabetes education, healthy eating, postprandial glycemic control

## Introduction

In the 20th century, dietitians were taught to formulate healthy meal plans for type 1 diabetes (T1D) patients. These plans provided a fixed amount of carbohydrates (CHO) from each meal and snack, based on the patient’s habits. This rigidity may have terrified some T1D patients. Nevertheless, it seemed logical to associate a fixed amount of CHO with a fixed amount of rapid-acting insulin. T1D patients were taught how to follow their plans—for example, how to take some potatoes off their plates to have a piece of bread instead.

Then, at the end of the 20^th^ century, functional insulin therapy (FIT) introduced an inverse paradigm. After observing meal compositions (especially CHO), pre- and postprandial glycemic profiles, and insulin doses, an insulin/CHO ratio can be defined: x international units (IU) of insulin/10 g of CHO (e.g., 0.8 IU/10 g) or 1 IU/x g (e.g., 1 IU/7 g). With the help of a dietitian, the T1D patient first counts the amount of CHO to be consumed and then calculates the required bolus of rapid insulin (1). This offers nutritional flexibility with real gains in quality of life and better diabetes parameters (2). Another recent example of T1D program is the UK’s Dose Adjustment for Normal Eating (DAFNE) (3).

These flexible methods still require self-discipline and are difficult to follow for individuals with a daily burden, such as T1D patients. Moreover, long-term benefits are not guaranteed (4) since they depend on permanent efforts on the part of T1D patients. These nutritional efforts focus on CHO counting because what matters is respecting the insulin/CHO ratio to achieve correct postprandial glycemic control. In this equation, healthy eating discipline often becomes of secondary importance. This omission could notably worsen postprandial glycemic control (5, 6).

Over the past few years, semi-closed-loop hybrid insulin pumps have been developed and are available for T1D patients, increasing flexibility and lowering the risk of hypoglycemia (7). What do these devices change in terms of T1D patients’ nutrition? What are the skills that dietitians should emphasize when teaching T1D patients using these pumps? This article offers an opinion on current and future nutrition education for T1D patients.

## Nutrition guidelines for T1D patients

The guidelines of the American Diabetes Association/European Association for the Study of Diabetes (7), and the Academy of Nutrition and Dietetics (2) highlight the importance of preventing cardiovascular (CV) events for T1D patients, which is the main goal of T1D nutrition education. They insist on a healthy diet through education tailored to each patient’s abilities, preferences, literacy and numeracy, prognosis, and cultural barriers, so that T1D patients can live the healthiest life possible and reduce the daily burden of this chronic disease (2, 7). They also recommend teaching T1D patients how to count CHO using tools selected according to each patient’s preferences (2). The three prisms of evidence-based practice—the scientific literature, therapists’ expertise, and patients’ values (8)—are obviously considered in these guidelines.

## How healthy eating patterns matter?

We know that T1D and non-diabetic patients with a history of CV events have the same risk of a CV event (9). This suggests that T1D could be considered a CV disease (CVD). We also know that adherence to a Mediterranean diet is an effective means of primary, secondary, and tertiary CVD prevention for the general population (10–12).

A study on adherence to a Mediterranean diet among T1D children reported very low adherence scores (13), in accordance with the general population (14). As nutrition specialists, we should not forget that healthy eating patterns—such as those associated with the Mediterranean and DASH (i.e. Dietary Approach to Stop Hypertension) diets—are not only the cornerstone of CVD prevention but also an efficient means of controlling postprandial glycemic values (5, 15, 16). Because it provides high amounts of fiber through non-starchy vegetables, fruits, pulses, whole grains, nuts and seeds, Mediterranean diet is logically the eating pattern mostly recommended for T1D patients (15, 17). Moreover, T1D patients—as well as the general population—should avoid added sugars and refined grains, and choose whole foods over highly processed foods (6, 17).

## The influence of fat and proteins on glycemic control?

According to the relevant guidelines, there is no ideal percentage of calories from CHO, fat and proteins for T1D patients. But meal composition should be addressed, as high amounts of fat and/or proteins can have—with significant differences among individuals—an impact on glycemic control. This impact may require adjustment of insulin dosage and/or duration of insulin delivery (7, 15).

Due to delayed gastric emptying and subsequent slower absorption of CHO, the presence of fat at a meal may improve postprandial glycemic control. But the quantity of fats, as well as the quality, can influence insulin sensitivity, which is particularly worsened by saturated fatty acids (18). In many studies, a large amount of fat (≥40g) increases insulin resistance and therefore requirements, but with clear differences among individuals. A strategy that appears to be effective is to split the insulin bolus into two different insulin delivery times (e.g. 10 minutes before and 1 hour after eating), to cover the extended postprandial period with increased insulin needs and to prevent early hypoglycemia (19–23).

This delayed hyperglycemic effect is also measured with high-protein meals, often during 3 to 5 hours and with clear differences whether or not the meal contains CHO. A high-protein meal (>40-75g) requires insulin, but especially if it does not contain CHO. The splitting of the bolus into two different insulin delivery times also seems effective with high-protein meals (19, 20, 22, 24).

It should be noted here that these high-fat (>40g) and/or high-protein (>40-75g) meals are not balanced and therefore not suitable for T1D patients on a daily basis. Dietitians should reinforce education in the principles of healthy eating, since a balanced meal is more manageable in terms of postprandial glycemic control. However, even if CHO remain the main determinant of postprandial glycemic control (6, 15), teaching how to deal with those special meals also takes part of the role of dietitians in T1D education.

## The role of exercise on glycemic control

Physical activity is known to improve fitness and CV health (25, 26). In T1D, it is associated with lower risk of microvascular complications and cancer (27). It is really effective in increasing insulin sensitivity and therefore lowering the insulin needs of the meals following exercise (25, 26). But to be safe, the risk of hypoglycemia should be mastered, notably with continuous blood glucose monitoring (CGM) controls and analysis.

## The beneficial use of CGM

CGM are the standard devices for glucose measurement and monitoring in T1D patients. It measures interstitial glucose to provide an estimation of plasma glucose and is able to alert the person in case of low or high glycaemia (7). They improve glycated hemoglobin and reduce the occurrence of hypoglycemia (28–30). They provide reports that could be analyzed retrospectively by T1D patients—with or without healthcare professionals—to adjust therapeutic strategies and behaviors, such as the influence of exercise on insulin requirements at following meals.

## Carbohydrates counting

With new T1D technologies (in particular semi-closed-loop hybrid insulin pumps), CHO counting is crucial for optimal glycemic control since only the patient can enter this information into the system. Neither CGM devices nor artificial intelligence can currently help in this counting. CHO counting is challenging for T1D patients (6, 7), and incorrect counting is the main identified cause of poor postprandial glycemic control (31).

Although CHO counting can contribute to better quality of life by improving nutritional flexibility (32) and to improved glycated hemoglobin (33, 34), complete accuracy is not necessary. A study involving T1D patients with controlled glycated hemoglobin showed that errors of ±10 g made no significant difference in postprandial glycemic control (35). On the other hand, a study showed that errors of 20 g caused a deterioration in postprandial glycemic control (36). It should then be kept in mind that a CHO counting accuracy of ±10 g is perfectly fine.

Interestingly, a study found that T1D patients overestimated most small meals and underestimated most big meals (31). We may thus hypothesize that T1D patients have a sort of insulin bolus standard and add or cut 1 or maximum 2 IU, with an insulin bolus range that does not reflect the real range of the CHO contents of different meals, which is very wide. Indeed, a balanced meal with vegetables, a lean source of proteins, and a standard starchy food contains 30–50 g of CHO, while a pizza may contain up to 150 g. The bolus should then increase from simple to quintuple, which few T1D patients dare to operate.

It is crucial to find easy and valid means for CHO counting, and dietitians should help T1D patients find the best tools for them. Let us first mention tables, including pictures, which present standard servings of CHO sources, then education programs such as FIT and DAFNE, and finally smartphone applications. The latter must contain pictures of servings, be easy to use, use valid nutritional value tables, and, if possible, be free of charge. The smartphone application market is growing rapidly, but few applications are reliable and helpful. Assessing an application developed in collaboration with patients, dietitians, and T1D educators and designed to be particularly easy to use, a 2020 study reported higher CHO counting accuracy and fewer errors of >10 g compared to usual care (37).

CHO counting is important, but we, as health professionals, must remember that T1D patients must make this stressful assessment at least three times a day. Therefore, we also have a duty to lighten their daily burdens and remember that there is no perfect tool since each patient has their own preferences.

## What do semi-closed-loop hybrid systems change for dietitians?

With the new technologies available, T1D patients must switch to counting CHO amounts from their meals, instead of calculating an insulin bolus. Therefore, T1D nutrition education places a greater emphasis on CHO counting, even if adherence to a healthy diet is generally poor. The risk of both dietitians and T1D patients forgetting the importance of a healthy diet increases, since CHO amounts are often the only information—with exercise—requested by the system. Although CHO counting can never be the only goal of T1D nutrition education, it is crucial for dietitians to be familiar with CHO counting tools. The easier to use an application is, the more it can help lighten the daily burdens of CHO counting.

The dietitian’s role basically remains the same: we must help patients control postprandial glycaemia using the entire range of our competences. We could propose some simple rules to T1D patients, such as entering every meal into the system, assessing CHO amounts with an accuracy of ±10 g, and following healthy eating patterns. Especially since the hybrid closed-loop systems do not prevent poor postprandial glycemic control with unbalanced meals, as those composed mainly of refined grains (6). Without forgetting the influence of fat and proteins on the prandial insulin requirements of certain particular meals, which must remain occasional.

## Conclusion

Dietitians should provide T1D patients with tailored education. They must be aware of all the possible means—nutritional and others—of achieving better postprandial glycemic control. CHO counting is an important means, but it is demanding for T1D patients. Dietitians should assume part of the responsibility of reducing T1D patients’ mental loads, by finding the most suitable tool(s) to count CHO for each patient. They should also be aware of the ineffectiveness of setting too rigid CHO counting objectives. The new T1D technologies available do not significantly change these considerations. However, they must not make us forget the importance of healthy eating patterns for postprandial glycemic control and CVD prevention, which are the two main objectives of T1D nutrition education. As dietitians, we should find a balance between the rigidly healthy meal plans of the 1980s and today’s junk food flexibility.

## Author contributions

The author confirms being the sole contributor of this work and has approved it for publication.
